# Tensiometry as a Simple Analytical Method for Quantification of Solubility and Release of Aroma Molecules in Aqueous Media

**DOI:** 10.3390/molecules26247655

**Published:** 2021-12-17

**Authors:** Ruth Kudla, Jochen S. Gutmann, Larisa A. Tsarkova

**Affiliations:** 1 Germain Textile Research Center North-West (DTNW), 47798 Krefeld, Germany; ruth.kudla@currenta.biz (R.K.); jochen.gutmann@dtnw.de (J.S.G.); 2 Physical Chemistry, University Duisburg-Essen, 47057 Duisburg, Germany; 3 Center for Nanointegration Duisburg-Essen (CENIDE), 45141 Essen, Germany; 4 Department of Chemistry, Moscow State University, 119991 Moscow, Russia

**Keywords:** essential oils, monoterpens, surface activity, solubility in aqueous media, dynamic tensiometry, fragrance carrier

## Abstract

Dynamic tensiometry is shown to be a high-potential analytical tool in assessing physico-chemical characteristics of fragrance molecules, such as solubility limit, volatility as well as much rarely assessed interfacial activity of these amphiphilic molecules. Surface tension of aqueous solutions of selected essential oils has been measured as a function of time and fragrance concentration using maximum bubble pressure method. The effect of the temperature and saline solution on the rate of dissolution in water was assessed. Dynamic surface tension turned to be sensitive to the composition of fragrances, as demonstrated on examples of natural and synthetic mixtures. Furthermore, presented work reveals the possibility of maximum bubble pressure tensiometry method to quantify the amount of fragrance compositions in flavored salts, including the artificially aged carrier samples. Suggested here analytical approach can be used for the detection of the purity of essential oils, for the optimization of compositions and of the manufacturing processes of fragrances-containing products, as well as for the assessment of the release/evaporation of fragrances from carrier systems.

## 1. Introduction

Perfumes, besides being noble products themselves, are often added as components to the products in cosmetic, textile, wellness or home-care branches in order to add a superior quality and thus to influence the consumer behavior. Accordingly, intensive research is dedicated to the understanding of the olfactory and other biological functions of perfume molecules [1,2]. In research and development of fragrances-containing products, odor assessment is usually performed by human olfactory system or by gas chromatography, as well as by chemo- or bio-electronic sensing devices [3,4,5].

Essential oils are generated through different biosynthetic routes and extracted from vegetable raw materials. The variety of odor compounds is represented by aliphatic or aromatic amphiphilic molecules with varied carbon backbones and diverse functional groups, including aldehydes, esters, ketones, alcohols, alkenes, carboxylic acids, amines. Recent research indicates, that rich and complex compositional variability of these poorly water soluble volatile amphiphiles defines their specific surface-active properties which can be assessed with tensiometry methods [6,7]. However, so far their intrinsic amphiphilic properties have not been thoroughly considered from the physico-chemical and analytical point of view, so that the research in this field is in its infancy.

Surface tension of aqueous solutions depends on the adsorption of a solute at the liquid–vapor interface [8]. This process, however, is time-dependent. Most studies of the surface tension focus on the equilibrium conditions, i.e., on the maximum surface coverage which is typically achieved on the time scale of tens of minutes to hours. More relevant to technological processing and, presumably, to the functions in biological systems is the dynamic surface tension, i.e., an ability of an amphiphile to decrease surface tension of aqueous solutions at a time scale of milliseconds and below [9,10,11]. A revealed distinctive feature of fragrance molecules, which can be considered as volatile amphiphiles, is their high dynamic interfacial activity, much higher than that of conventionally used technical surfactants [6]. Another potentially useful property of such amphiphiles is their volatility, so that they notably evaporate from interfaces. Accordingly, the surface tension of their aqueous solutions increases on a time scale of minutes. Monitoring the increase of the surface tension allows evaluating of the evaporation rate and of the material constant, which turned to be specific to the chemistry of the aroma molecules [12]. Further development of the proposed analytical approach concerns the possibility to evaluate molecular interactions of aroma molecules with other components of complex formulations, such as surfactants, polymers, salts and pigments [13], as well as the release of fragrances from carrier systems, which typically represent multicomponent, heterogeneous systems such as emulsions [14,15,16], foams [17], capsules [18], liposomes [19,20], latexes [21], surfactant solutions [13,22,23,24] or from smart cleavable surfactant profragrances [25].

In this work dynamic tensiometry, i.e., the assessment of the time evolution of the surface tension, is shown to be a useful analytical tool in evaluating quantitative physico-chemical characteristics of fragrance molecules, such as solubility limit, volatility, and interfacial activity. We first present surface tension of aqueous solutions of selected essential oils, which has been measured continuously during saturation process to determine the solubility limit. Then we discuss the sensitivity of the surface tension to the fragrance concentration, the composition of natural and synthetic mixtures as well as to external factors such as water salinity and temperature. Finally, the possibility of the maximum bubble pressure method to quantify the amount of fragrance molecules in flavored salts, including artificially aged carrier samples, is demonstrated. This method can be considered as a valuable complementary analytical tool to assess structural and compositional heterogeneity, chemical aging, molecular interactions of aroma molecules in technologies related to isolation and usage of essential oils and fragrances.

## 2. Results and Discussion

### 2.1. Dynamic Surface Tension of Saturated Solution of Essential Oils

**Figure 1:**
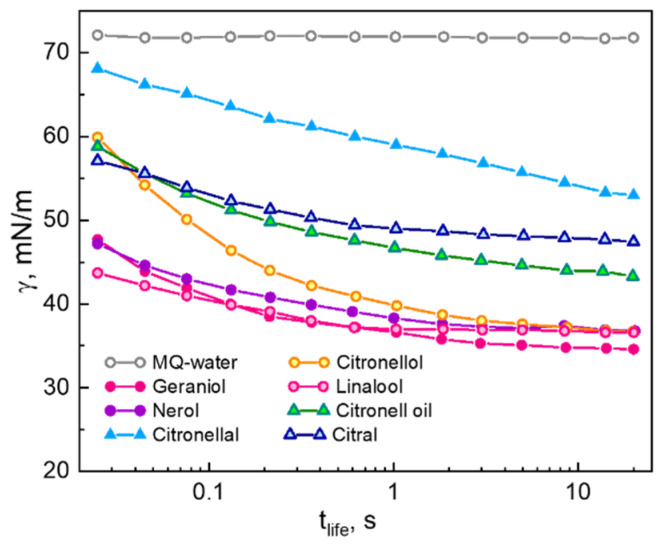
Surface tension versus surface lifetime (*t_life_*) of water and of saturated solutions of indicated essential oils/aroma molecules (Table 1).

Figure 1 shows the curves of the surface tension versus life-time (*t_life_*) of saturated solutions of indicated fragrances, among others of monoterpene alcohols, which are typically poorly soluble in water (Table 1). As clearly seen, almost all studied solutions already in a millisecond range exhibit a significant reduction of the surface tension as compared to that of pure water, which is time-invariant. The rapid reduction of the surface tension of water is an indication of the fast diffusion and adsorption of a solute at water-air interface. We note that this fast adsorbing behavior is a remarkable feature of poorly soluble aroma molecules as compared to conventional technical surfactants, as well as to the solutions of highly soluble short-chain alcohols [6].

Each measured curve exhibits a decrease in the surface tension with increasing the surface life-time (*t_life_*), until time-invariant values are reached, e.g., for monoterpene alcohols geraniol, nerol and linalool. As also seen in Figure 1, the surface activity of alcohols is higher than that of fragrance compounds with an aldehyde group (citronellal and citral) since the former provide a more fast and effective decrease of the surface tension. This effect cannot be entirely attributed to the respective solubility of the aroma molecules in water, i.e., to the concentration of the measured saturated solutions (Table 1). Saturated solutions of the monoterpenes nerol, geraniol and linalool show similar kinetics of the surface tension, despite large differences in solubilities (1.45 g/L for linalool and practically insolubility for nerol).

Furthermore, saturated solution of insoluble in water citronella oil, which is a natural multicomponent mixture of geraniol, citronellal and citral (Table 1), is also characterized by a reduced surface tension already in the millisecond range.

These examples, on one side, demonstrate molecular-structure defined differences in the amphiphilicity and, accordingly, in the surface activity of the aroma molecules, which can be systematically studied using low-cost and high throughput tensiometry methods. On the other side, this analytical method can be used to verify available in data-bases values of molecular solubility of essential oils. Establishing “Structure-property relationship” of such aroma molecules with regards to their molecular structure, volatility and interfacial activity is envisaged to be an exciting and application-relevant research direction where an interdisciplinary collaboration of synthetic, analytical and biochemistry scientists may result in new insights and applications. Importantly, the concentration of a fragrance in the interfacial layer is directly linked to its concentration in the vapor phase, which suggest a possibility to correlate tensiometric and head-space chromatography measurements.

It should be noted, that the measurement geometry of the MBP method (a small-volume air bubble in a large-volume solution) is particularly suitable for the assessment of the interfacial behavior of volatile amphiphiles in comparison with static (equilibrium) tensiometric methods. In Pendant drop or Wilhelmy Plate tensiometry methods an exposure of extended interface to the environment may result in the uncontrolled evaporation of the volatile amphiphile, causing the surface tension to increase with time [26].

We believe that high interfacial activity of perfumes in aqueous solutions can be attributed to the existence of mesoscale solubility [27]. According to Rak and Sedlak [27], mesoscale structures in aqueous solutions of organic liquids are not-fluctuating objects with macroscopic lifetimes and near-spherical shapes with radii in the range 30−300 nm. These aggregates provide a high local concentration of a solute, also in the vicinity of the interface. Accordingly, an increase an effective diffusion coefficient results in the observed fast-adsorbing behavior.

### 2.2. Kinetics of Solution Saturation under Variation of the Temperature and Presence of the Salt

**Figure 2:**
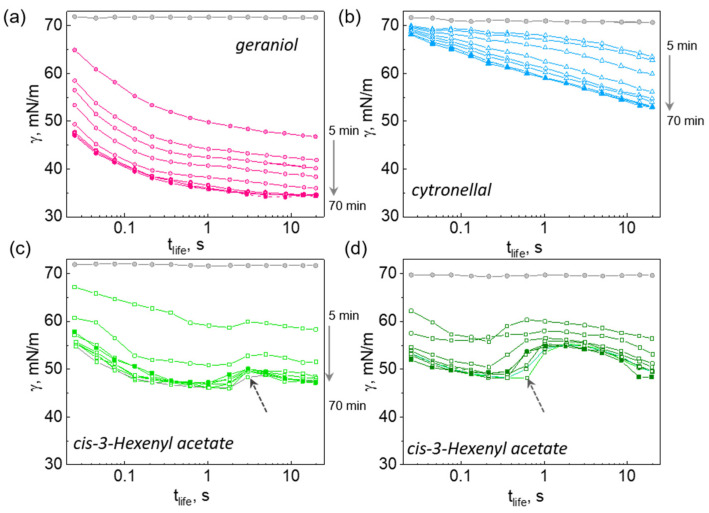
Dynamic curves of the surface tension versus surface life-time (*t_life_*) measured at 25 °C (**a**–**c**) and at 40 °C (**d**) for water (gray curves in all plots), geraniol (**a**), citronellal (**b**) and cis-3-Hexenyl acetate solutions during the saturation process. Shown curves have been measured after 5 min up to 70 min of saturation with 5 min intervals. Gray dashed arrows in (**c**,**d**) point to the curves taken at 25 min saturation time. Solid symbols in each plot indicated solutions after 70 min of saturation.

Saturated solutions of the studied aroma molecules have been prepared by adding an excess of the organic phase to water followed by continuous stirring to achieve saturation with molecularly dissolved aroma molecules. The dissolution process has been monitored by taking an aliquoted of the water phase in 5 min intervals for the surface tension measurements. As exemplarily shown in Figure 2a for geraniol solution, the shape of the kinetic curves does not change with the saturation time, with the upper curve taken after 5 min and the lower curve (solid symbols) measured after 70 min of saturation being similar. The curves just appear shifted along the Y-axis to the lower values of the surface tension. The decrease of the surface tension is due to the growth of the molecular concentration in solution, which simultaneously leads to a higher adsorption at the air-water interface and. Before each measurement, the solutions were filtered through a polyether sulfone filter (with a pore size of 500 μm) to remove excess oil droplets from the solution and to achieve a good reproducibility of the surface tension measurements using MBP method.

The dynamic surface tension of solutions of citronellal (Figure 2b) differ from that of geraniol both in the shape of the curves versus surface life time (*t_life_*) and in their evolution with the saturation time. In particular, the drop of the surface tension in the ms range is not so pronounced as for monoterpene alcohols, and the equilibrium (time-independent) values of the surface tension are not achieved in a lapse time of 10 s of the measurement. This observation suggests a strong diffusion-limited adsorption process of citronellal molecules at the surface.

Shown in Figure 2c,d is the dynamic surface tension of solutions of cis-3-Hexenyl acetate during saturation and measurement at 25 °C (Figure 2c) and 40 °C (Figure 2d). The non-trivial shape of the curves can be explained by a presence of solutes with different diffusivity and interfacial activity, i.e., the kinetic curves are indicative to the purity/composition of the oil. The initial steady decrease of the surface tension up to *t_life_* of ca 0.4–0.5 s and ca 2 s in Figure 2c,b, respectively, is due to adsorption of a faster and a more surface-active component, which is on a longer time-scale is replaced from the interfacial layer by a slower/less surface-active component. This replacement may be the result of a faster evaporation behavior of the former. Another possible explanation, is the “reaction”- driven interfacial behavior [28], when adsorbed molecules of *cis*-3Hexenyl acetate undergo a phase transition from molecular to condensed state, resulting in a “mosaic”-like structure within the interfacial layer. This may lead to an effective increase of the area per molecule, and hence to temporal increase of the surface tension, which returns to the low value as more molecules reach the interfacial layer.

Interestingly, the time-scale of the surface tension modulation appears to be dependent both on the temperature of the dissolution/measurements (compare Figure 2c,d), and on the kinetics of the dissolution/extraction into the water phase. As indicated by dashed arrows in Figure 2c,d, the lowest surface tension at *t_life_* of about second is achieved not for a saturated solution, but for a solution which was extracted in 25 min. On the other hand, at a time scale of tens of seconds, the lowest surface tension is exhibited by fully saturated solutions. It should be noted, that since aroma molecules can desorb from the interface into the gas phase, tiny differences in the values of the saturated vapor pressure may affect the composition of the interfacial layer, i.e., the values of the surface tension. A quantitative evaluation of such non-trivial behavior of the dynamic surface tension is beyond the scope of the current study.

Generally, a calibration curve (isotherm) can be generated and used to determine unknown concentrations of a solute in test solutions [6,26]. The concentration at which the minimum value of the surface tension is achieved according to the calibration curve (isotherm) presumably indicates the limit of the molecular solubility of the investigated substance. This data can be and compared with the solubility data, which is available in literature (Table 1). The discrepancy observed in our study (e.g., for citronella oil and nerol) presumably can be explained by the existence of the mentioned above “meso-aggregates” of organic liquids in water [27]. These aggregates may lead to the fast-adsorbing behavior at the interface.

**Figure 3:**
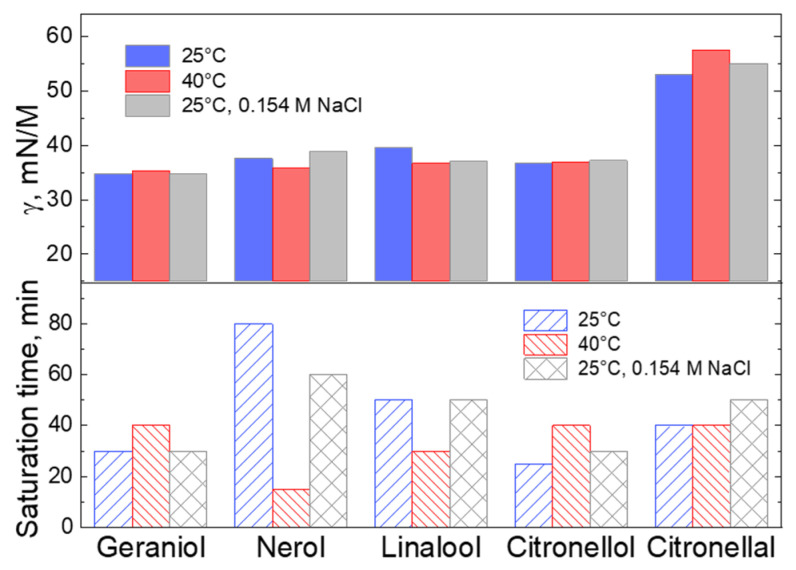
Summary of the surface tension at a *t_life_* of 20 s (**top** panel) and of the dissolution time (**bottom** panel) for indicated substances, evaluated at 25 °C in Milli-Q water and in salinity solution (0.154 M NCl) and at 40 °C.

Figure 3 summarizes measurements, which are presented in Figure 2a,b for geraniol and citronellal, and for some other selected essential oils. It displays for indicated substances the surface tension at *t_life_* of 20 s, as well as the time needed to achieve saturation as a function of the temperature of the saturation/measurement and of the presence of salt (physiological solution). While the quasi-equilibrium surface tension of monoterpene alcohols is more or less invariant to the molecular structure and solution temperature/presence of salt, as follows from the strong deviations in the saturation time, the diffusion and adsorption activity is clearly dependent on the molecular and environmental features. These observations are in agreement with the earlier studies, which emphasized the importance of molecular mobility (translational diffusion and mass transfer through phases) of volatile compounds on their release from the matrix [29].

**Figure 4:**
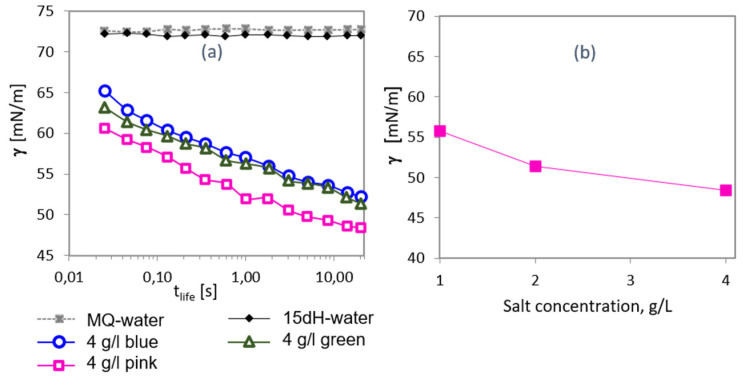
(**a**) Surface tension of water (grey symbols), synthetic water with a water hardness of 15° dH (black symbols) and of solutions of three flavored salts each with a concentration of 4 g/L (pink, green and blue symbols, respectively) versus surface life-time (*t_life_*). (**b**) Surface tension (at 20 s surface life-time) of pink salt solutions versus concentration in the solution.

Although the understanding of scientific basics behind these differences (or similarities) in the interfacial adsorption of aroma molecules is still to be elaborated, the data in Figure 3 and Figure 4 clearly shows that tensiometric measurements have a high sensitivity with regards to the concentration as well as to cumulative compositional heterogeneity of essential oils in aqueous solutions. We believe that combining tensiometry with other analytical methods, which quantify the molecular structure of the solutes, will advance the methods of isolation, purification and characterization of plant-based substances.

### 2.3. Assessment of the Fragrance Load and Carrier Aging

Demonstrated surface activity of aroma molecules was used to analyze flavored salt products, which apparently differ in color and in composition of loaded fragrances. Figure 4a shows kinetic curves of the solutions of pink, green and blue salts, as well as the surface tension of water with a hardness of 15° dH. While it is well known that salts do not decrease the surface tension of water, the dissolved fragrances have a clear influence on the surface tension. We note, that flavored salt apparently has been loaded with different fragrance compositions, so that the solutions and interfacial layers contain mixtures of aroma molecules rather than individual substances. As exemplary shown in Figure 4b for a pink flavored salt, the higher the concentration of the dissolved fragrance carrier, the lower is the surface tension at 20 s *t_life_*, i.e., the higher is the adsorbed amount of fragrances in the interfacial layer.

**Figure 5:**
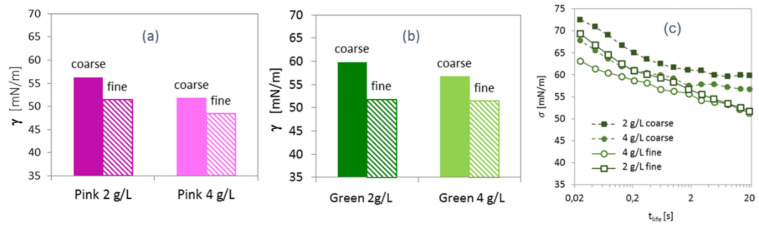
(**a**,**b**) Surface tension (at 20 s surface-life time) of the solutions of coarse and fine fractions of pink (**a**) and green (**b**) salts with concentrations of 2 g/L and 4 g/L. (**c**) Surface tension versus surface-life time (*t_life_*) of solutions of coarse and fine fractions of the green salt with a concentration of 2 g/L and 4 g/L.

The examined salts have been fractionated into coarse and fine (less than 1 mm) fractions. Figure 5a,b shows the surface tension at 20 s *t_life_* of the solutions of pink and green salts as a function of the salt concentration in the solution for fine and coarse fractions. It can be seen, that dissolving of the fine salt fraction leads to a stronger decrease in the surface tension. This observation implies that in this case a larger amount of fragrance is released in the solution. Since the fine fraction has a larger surface area than a coarse fraction of the same mass, it can be concluded that the fragrances are loaded onto the surface of the salts grains.

Additional information can be gained from the consideration of the dynamic curves of the surface tension, which are presented in Figure 5c for green salt solutions. Increasing the amount of the dissolved coarse fraction from 2 to 4 g/L leads to the increase of the dissolved fragrance composition in solution, as reflected in a lower surface tension of the latter solution. However, dissolving a larger amount of the fine fraction of the green salt (4 g/L) does not lead to a further decrease in the surface tension (at 20 s surface life-time) as compared to the solution with 2g/L salt concentration. Although these two curves show clear differences in the range of *t_life_* below second, the amount of the loaded fragrance, presumably, exceeds the solubility limit in water, so that the excess of fragrances in 4 g/L solution is likely dissolved in a form of mesoscale oil droplets.

**Figure 6:**
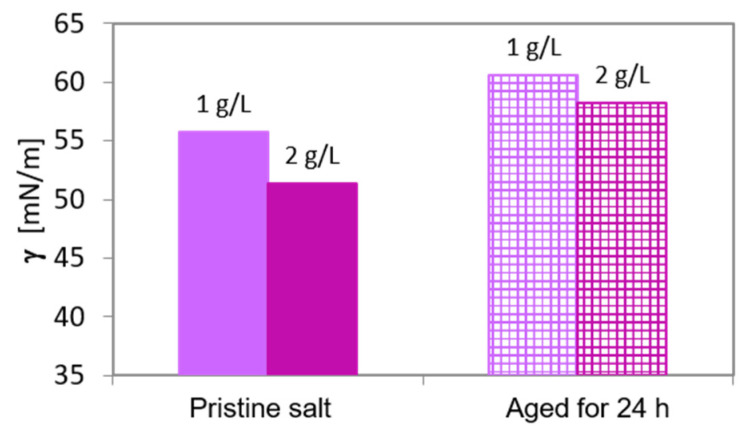
Surface tension (at 20 s surface life-time) of pink salt solutions with a concentration of 1 and 2 g/L before and after artificial aging for 24 h.

Such simple and high throughput tensiometry method can also be used to evaluate the ageing of products containing aroma molecules. To demonstrate this, the pink salt was artificially aged by storing under exhaust snorkel with a defined exhaust air rate of 1 m^3^/h for 24 h. Figure 6 shows the surface tension of the solutions of the original and of the artificially aged pink salt. The surface tension of the solutions from the aged salt is higher than that of respective solution from the fresh product. As mentioned above, by establishing a calibration curve (isotherm) of the used fragrance composition it is possible to evaluate quantitative information regarding the concentration decrease (and respective loss of the product quality) and accordingly to develop recommendations on the optimal storage of the product.

## 3. Materials and Methods

Aroma molecules (Table 1) have been provided by NHU Europe GmbH (Bardowick, Germany). Flavored salts with pink, blue and green colors (DPC spa, Gorla Minore, Italy) have been used as received. Solutions have been prepared using Milli-Q purified water or 0.9% NaCl (from Aldrich, St. Louis, MO, USA) solution.

**Figure 7:**
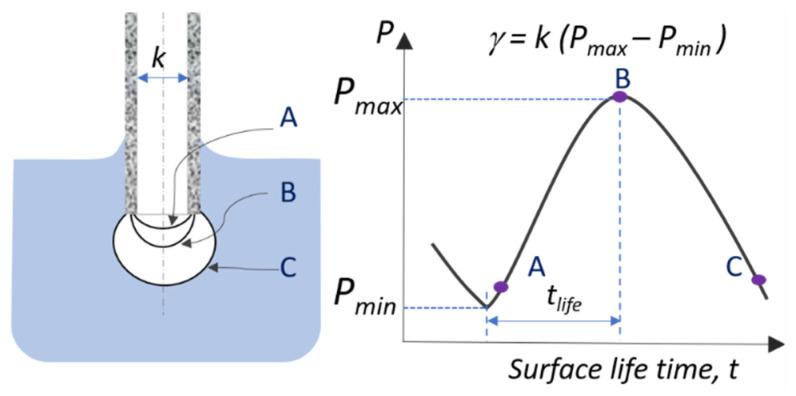
Schematic representation of the measuring principle of the maximum bubble pressure (MPB) method for the measurements of the dynamic surface tension γ of aqueous solutions. The surface tension is calculated from the measurement of the pressure difference (*P_max_ − P_min_*) in the bubble in a capillary with a known radius 1/2k, as indicated.

Surface tension γ was measured using maximum bubble pressure (MBP) tensiometer “Sita pro line t100” (SITA GmbH, Dresden, Germany) with a capillary made of polyether ether ketone (PEEK). With this method dynamic surface tension at air-water interface can be determined as a function of the age of a newly formed surface with great accuracy from a few tenths of a millisecond to hundreds of seconds. Air bubble is formed at the end of the capillary, which is immersed in a testing solution, and its internal pressure changes continuously as the radius grows (see Figure 7). The surface tension is calculated from the difference between the maximum and minimum pressures in each air-bubble formation process. We note that surface active molecules (surfactants) are typically characterized at equilibrium adsorption conditions (at infinite surface age) using so-called static tensiometry methods.

Saturated solutions of the studied aroma molecules have been prepared by adding an excess of the organic phase to MilliQ-purified water followed by continuous stirring using a magnetic stirrer to facilitate the diffusion of aroma molecules. Measurements of the surface tension have been performed at temperatures of 25 ± 0.5 °C and at 40 ± 0.5 °C.

## 4. Conclusions

Demonstrated here surface tension measurements expand the tool of analytical methods, traditionally applied to characterize fragrances. Using maximal bubble pressure method it is possible to assess physico-chemical characteristics of fragrance molecules, such as solubility limit, volatility as well as the interfacial activity of these amphiphilic molecules. Establishing “structure–property relationship” of such aroma molecules with regards to their molecular structure, volatility and interfacial activity is envisaged to be an exciting and application-relevant research direction. Importantly, the concentration of a fragrance in the interfacial layer is directly linked to its concentration in the vapor phase, which suggest a possibility to correlate tensiometric and head-space chromatography measurements.

As an application example, aqueous solutions of three differently colored carrier salts with different loaded fragrance compositions have been studied using dynamic tensiometry. The fraction of the carrier (coarse/fine) and the amount of the dissolved flavored salts has been varied. Additionally, the effect of the artificial aging on the amount of the loaded aroma molecules has been revealed. The demonstrated approach can be used for the optimizations of compositions and of the manufacturing processes of fragrances-containing products, as well as for the assessment of the release/evaporation of fragrances from the products.

Further development of the proposed approach will make it possible to evaluate the molecular interactions of aroma substances with other components of detergents and of cosmetic formulations, such as surfactants, polymers, salts and pigments.

## Figures and Tables

**Table 1:** Physicochemical properties of studied aroma molecules.

Volatile Amphiphile	Purity (GC)%	Molecular Weight, g/moL	Solubility in Water at 20 °C g/L	logP^b^ at 20 °C	Boiling Point, °C
Geraniol 98	98.7	154.25	0.686	3.28	229–230
Nerol	98.9	154.25	---	3.56	224–225
Linalool	98.8	154.25	1.45	2.44	198–200
Citronellol	96.8	156.27	-	3.91	224
Citronellal	98.8	154.25	0.07	3.53	208
Citral	97.8	152.23	0.42	2.33	225
Citronell oil	Geraniol (25–45%) and Citronellal (25–54%), Citral, Eugenol and Vanillin	154.25	---	---	208–230
*Cis*-3Hexenyl acetate	98.6	142.20	11.1	1.77	172

## Data Availability

Not applicable.

## Sample Availability

Samples of the compounds are not available from the authors.
